# Characterization of Novel Di-, Tri-, and Tetranucleotide Microsatellite Primers Suitable for Genotyping Various Plant Pathogenic Fungi with Special Emphasis on Fusaria and *Mycospherella graminicola*

**DOI:** 10.3390/ijms13032951

**Published:** 2012-03-06

**Authors:** Ali H. Bahkali, Kamel A. Abd-Elsalam, Jian-Rong Guo, Mohamed A. Khiyami, Joseph-Alexander Verreet

**Affiliations:** 1 Botany and Microbiology Department, College of Science, King Saud University, P. O. Box: 2455, Riyadh 1145, Kingdom of Saudi Arabia; E-Mail: abahkali@ksu.edu.sa; 2 Agricultural Research Center, Plant Pathology Research Institute, Giza, Egypt; 3 Institute of Phytopathology, Christian-Albrechts-University Kiel, Hermann-Rodewald-Str. 9, D-24118, Kiel, Germany; E-Mail: javerreet@phytomed.uni-kiel.de (J.-A.V.); 4 King Abdulaziz City for Science and Technology (KACST), P. O. Box 6086, Riyadh 11442, Kingdom of Saudi Arabia; E-Mail: mkhiyami@kacst.edu.sa; 5 Institute of Environment and Plant Protection, Chinese Academy of Tropical Agricultural Sciences, 71737 Danzhou, Hainan, China; E-Mail: guojianrong@hotmail.com (J.-R.G.)

**Keywords:** genotyping, tetranucleotide microsatellites, SSR, fungal plant pathogen

## Abstract

The goals of this investigation were to identify and evaluate the use of polymorphic microsatellite marker (PMM) analysis for molecular typing of seventeen plant pathogenic fungi. Primers for di-, tri-, and tetranucleotide loci were designed directly from the recently published genomic sequence of *Mycospherlla graminicola* and *Fusarium graminearum*. A total of 20 new microsatellite primers as easy-to-score markers were developed. Microsatellite primer PCR (MP-PCR) yielded highly reproducible and complex genomic fingerprints, with several bands ranging in size from 200 to 3000 bp. Of the 20 primers tested, only (TAGG)4, (TCC)5 and (CA)7T produced a high number of polymorphic bands from either *F. graminearum* or *F. culmorum*. (ATG)5 led to successful amplifications in *M. graminicola* isolates collected from Germany. Percentage of polymorphic bands among *Fusarium* species ranged from 9 to 100%. Cluster analysis of banding patterns of the isolates corresponded well to the established species delineations based on morphology and other methods of phylogenetic analysis. The current research demonstrates that the newly designed microsatellite primers are reliable, sensitive and technically simple tools for assaying genetic variability in plant pathogenic fungi.

## 1. Introduction

Microsatellites, also known as simple sequence repeats (SSRs) are tandemly repeated DNA sequence elements. They consist of repeats of 1–13 base pairs (bp) [1,2] which can be multiplied more than 100 times in a genome. They are highly abundant and present high levels of polymorphisms throughout all eukaryotic genomes and also in prokaryotic genomes [3]. Due to their high mutability, SSRs are regarded to play an important role in genome evolution [4].

Microsatellites or SSR markers have proved highly useful for molecular identification and differentiation among individuals and populations by PCR-based methods because of their frequently variable loci in the genome. The microsatellite-primed polymerase chain reaction (MP-PCR) uses single primers to produce DNA fingerprint profiles, requiring only small amounts of DNA for direct PCR and gel electrophoresis analysis [5]. In comparison with other marker techniques such as AFLP, RAPD, and RFLP, it is a quite simple and powerful fingerprinting tool due to its high reproducibility, ease of scoring, high throughput and codominance as well as to the high polymorphism of band profiles even among related species and genera [6,7]. Additionally, the amenability of SSRs to PCR amplification and relatively easy scoring make them valuable for analyzing large populations in different laboratories, which is not possible using RAPDs (randomly amplified polymorphic DNA) or AFLPs (amplified fragment length polymorphism) [8].

Fungi are the largest eukaryotic group in nature, including mushrooms, rusts, smuts, puffballs, truffles, morels, molds, and yeasts, as well as many less well-known organisms [9]. About 70,000 species of fungi have been described and approximately 1.5 million species may exist [10]. Like other eukaryotic organisms, fungi also contain abundant SSRs in their genomes [11]. The abundance and high variability of microsatellites allow their use as highly preferred markers to study the genetic diversity of various fungal species [12]. Recently, MP-PCR has been frequently and extensively applied for various fungal molecular biological studies such as genetic diversity [13–16], pathogen identification and differentiation [17] and fungal evolution [18].

Microsatellites have been, utilized in studying variability of fungi [13] such as *Ascochyta rabei* [19,20], *F. oxysporum* f. sp. *ciceri* races [14], *Fusarium oxysporum* f. sp. *radicis-lycopersici*, *Fusarium graminearum* [21], *F. poae* [22], *Fusarium* spp. isolates [23,24], *Phoma tracheiphila* [25,26]*. Pythium* [27,28] and *Rhizoctonis solani* [16,29,30].

In spite of approximately 40 microsatellite primers have been developed for separation of individual fungal species by MP-PCR, only a few have been proved to be applicable for a wider range of fungal species. Development of new microsatellite primers is therefore desirable for fungal molecular studies. Recently, Karaoglu *et al.* [11] analyzed and compared the occurrence and frequency of SSRs in nine complete fungal genomes. Their achievements offer a possibility to design new microsatellite primers suitable for multiple fungi based on the relative abundance of SSRs in genomes. In this study, we designed 20 new microsatellite primers based on SSRs of several fungal species reported by Karaoglu *et al.* [11] and tested their availability using 16 common fungal species.

## 2. Experimental Section

### 2.1. Culture of Fungal Species and DNA Extraction

Nineteen isolates of *Mycosphaerella graminicola* and 16 isolates representing other fungal species originating from different geographic locations (Table 1) were used to test the suitability of the tested primers. *Pyrenophora teres* was kindly provided by Dr. F. Maier in Biocenter Klein Flottbek, University of Hamburg and *P. tritici-repentis* by Prof. Dr. Holger B. Deising, Institut für Pflanzenzüchtung und Pflanzenschutz, University of Halle, Germany. The other isolates were obtained from Institute of Phytopathology, University of Kiel, Germany. *M. graminicola* was cultured in a malt-yeast-extract broth and the other species in potato dextrose broth on a shaker at 22 °C, 120 rpm*. M. graminicola* was harvested after 5 days by centrifugation at 8000 *g* for 2 min and washed once with sterile distilled water. The other isolates were harvested after 5–10 days by filtration and the water residue was removed with sterile filter paper. Spores or mycelia were ground to a fine powder in liquid nitrogen using a mortar and pistil and DNA was extracted using the method described by Cenis *et al.* [31] with minor modifications.

### 2.2. Primer Design

Seventeen repeated motifs were selected to design new primers based on their abundance in eight fungal genomes by analyzing in the SSR database generated by Karaoglu *et al.* [11]. Genome sequence data of *F. graminearum* (36.2 Mbp size) available from Broad Institute (www.broadinstitute.org) and the genome sequence of *Mycosphaerella graminicola* (39.7 Mbp*)* available at *Mycosphaerella graminicola* v2.0, (http://genome.jgi-psf.org/Mycgr3) were used to search for mono-, di-, tri-, and tetranuclotide motifs. The aim of this search was to explore repetitive sequences that were expected to have a very high degree of polymorphism. Altogether the twenty primers listed in Table 2 were designed. The suitability of primers was tested with 19 isolates of *Mycosphaerella graminicola* and 16 isolates representing other fungal species (Table 1).

### 2.3. Microsatellite Primed PCR

PCR mixtures contained 10 pmol primer; 10 mM Tris-HCl (pH 9.0), 50 mM KCl, 0.1% (v/v) Triton^®^ X-100, 2.5 mM MgCl_2_, 200 μM of each dNTP, 1 U of *Taq* polymerase and 5 ng of template DNA in a total volume of 25 μL. To obtain the optimal annealing temperature of each primer, a PCR was performed with 12 different annealing temperatures, *i.e.*, 40.1 °C, 40.5 °C, 41.3 °C, 42.7 °C, 44.4 °C, 46.4 °C, 48.3 °C, 50.3 °C, 52.6 °C, 54.0 °C, 54.7 °C and 55.2 °C in a Px2 gradient PCR thermal cycler (Thermo Hybaid, Ashford, United Kingdom). The reactions were carried out in a PTC-200 Thermocycler (MJ Research) according to the following PCR programm: 94 °C for 2 min, followed by 40 cycles of 94 °C for 1 min, 40–55°C (depending on primers used) for 90s and 72 °C for 2 min with a final extension at 72 °C for 6 min. PCR amplification with each primer was repeated at least twice to check the reproducibility of DNA profiles.

### 2.4. Visual Analysis of Banding Patterns and Gel Documentation

All PCR-amplified products were separated by electrophoresis on 1.5% agarose gels for 1.5 h at 7.0 V/cm^2^. After that, the gel was photographed using the GelSystem Flexi gel documentation system (Biostep, Jahnsdorf, Germany). The banding patterns obtained were compared for polymorphism by visual observation. Visible bands among isolates with the same migration distance were considered the same. Each distinct band was considered as polymorphic band and was scored for the presence (1) or the absence (0) among the isolates. Only reproducible bands in repeated PCR amplification were considered for analyses. A dendogram was computed (Gene Tools by SynGene) using the DICE similarity coefficient and Unweighted Pair Group Method with Arithmetic Mean (UPGMA).

## 3. Results

Based on the abundance of SSR in eight fungal genomes in the SSR database Karaoglu *et al.*, [11], 20 repeat motifs were selected to design new primers. Altogether 20 microsatellite primers were designed (Table 2). To get the optimal annealing temperature of each primer, a PCR was performed with 12 different annealing temperatures ranging from 40–55 °C (Table 2). The results showed that annealing temperature had different influence on these primers. For (GTCA)4, (TTTC)4, no clear difference were observed between the banding patterns produced at different annealing temperatures. (TGAC)4 amplified clearer banding pattern at higher temperatures, whereas (GTA)5, (TAC)5, (TGC)5, (TGT)5 and (GGTT)5 gave the best banding patterns at 40 °C. For (CT)7G, (AG)7C and (TAGG)4, the optimum temperature was 48 °C, at which clearer fingerprints with a higher number of bands was produced as compared with those at other annealing temperatures.

**Figure 1:**
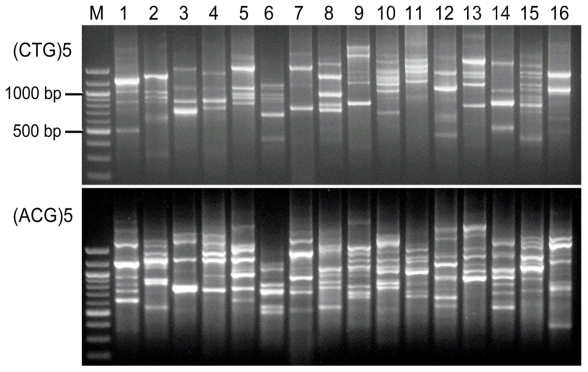
DNA fingerprinting profiles amplified from 16 different common phytopathogenic fungal species using the two newly designed primers (CTG)5 and (ACG)5. Twenty μL of PCR products were separated by electrophoresis on a 1.5% agarose gel for 1.5 h at 7.0 V/cm^2^. Lanes 1 to 16 are *Fusarium oxysporum f. sp. vasinfectum*, *F. solani*, *F. graminearum*, *F. culmorum*, *F. poae*, *Macrophomina phaseolina*, *Trichoderma harizinum*, *Stagonospora nodorum*, *Septoria tritici, Pyrenophora tritici-*repentis, *P. teres*, *Pseudocercosporella herpotrichoides*, *Penicillium* sp., *Alternaria* sp., *Cercospora beticola*, *Chaetomium* sp. M is the 100-bp DNA ladder.

The suitability of these primers for typing fungi was tested with 16 common fungal species in comparison with five previously established primers. All the primers tested produced banding patterns for at least one fungal species. The sizes of fragments amplified ranged mostly from 300–2000 bp (Table 2). Of these primers, (CA)7T, (CTG)5, (AGG)5, (TCC)5, (ACG)5, (TGG)5, (ATG)5, (TGAC)4, (GTCA)4, (TTTC)4 and all the five previously established primers amplified clear banding patterns from all the 16 species tested (Table 3). (AGG)5 and (ACG)5 generated 107 and 103 bands, respectively, from these species, with 6.7 and 6.3 for each species (Figure 1). (AG)7C gave rise to clear fingerprints from 15 of the 16 species except *S. nodorum*, (TAGG)4 except from *Chaetomium* sp. Primer (CT)7G amplified no band from *M. phaseolina* and *C. beticola*, and (GTA)5 did not amplify DNA from 10 out of 16 species. (GGTT)5 led to five clear bands from *S. nodorum* and *Alternaria* sp., but it is not optimal for other species tested. Although some bands were amplified from some of the species by (TGC)5, (GCT)5 and (TGT)5, these primers produced smear banding patterns and led to an unclear background. (TAC)5 amplified three bands only from *S. tritici*.

**Figure 2:**
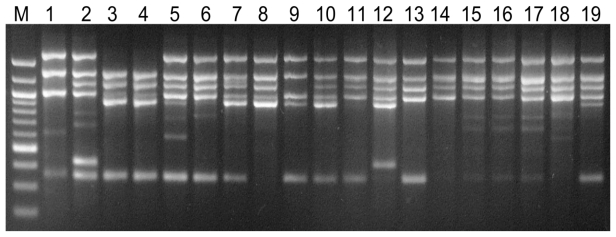
DNA fingerprinting profiles amplified from 19 different single-spore isolates of *Mycosphaerella graminicola* using a newly designed primer (ATG)5. Lanes 1 to 19 are the isolates K-Or-1, K-Or-30, K-Or-38, K-Or-44, OK-102, OK-108, OK-109, OK-112, OK-120, K-Ba-10, K-Ba-20, K-Ba-30, K-Ba-40, K-Ba-60, G-Or-1, G-Or-6, G-Or-8, G-Or-88 and G-Or-102 of *M. graminicola*. M is the 100-bp DNA ladder.

**Figure 3:**
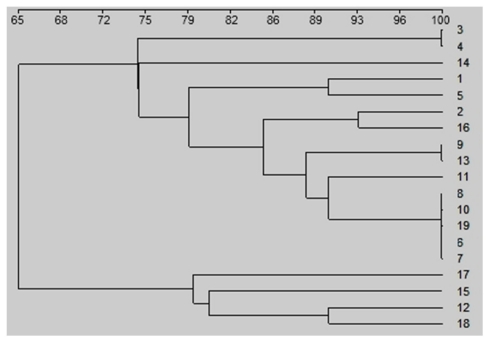
Dendrogram based on the Dice coefficient constructed by unweighted pair-group method with arithmetic average cluster analysis of 19 different single-spore isolates of *Mycosphaerella graminicola* using a newly designed primer (ATG)5. Positions 1 to 19 are the isolates K-Or-1, K-Or-30, K-Or-38, K-Or-44, OK-102, OK-108, OK-109, OK-112, OK-120, K-Ba-10, K-Ba-20, K-Ba-30, K-Ba-40, K-Ba-60, G-Or-1, G-Or-6, G-Or-8, G-Or-88 and G-Or-102 of *M. graminicola*.

Almost all the primers that led to clear multiple banding patterns also showed a high polymorphism among different fungal species. We compared the production of polymorphic bands by these primers between two genetically closely related *Fusarium* species (Table 3). Of the 20 primers tested, only (TGG)5, (GTA)5 and (GGTT)4 produced no polymorphic bands from either *F. graminearum* or *F. culmorum*. (TAGG)4, (TCC)5 and (CA)7T produced a high number of polymorphic bands from the two species, with 8, 7 and 5 polymorphisms, respectively. (AG)7C, (TTTC)4 and (TGT)5 amplified 100% of polymorphic bands, with 4. 4 and 1.0 bands, respectively (Table 4). Using the Gene Tools software package and the Dice coefficient, trinucleotide microsatellite primer (ATG)5 amplified genomic DNA fragments from all sixteen isolates and clear distinction of the isolates was possible (Figure 2). Genetic similarity of the isolates was determined using the Dice’s coefficient and the UPGMA clustering method. Two major groups were observed in the resulting dendrogram, which was divided into three subgroups. Between *M. graminicola* isolates, similarities ranged from 74 to 100% for intraspecific comparisons (Figure 3). The results showed that isolates 3 and 4 had the highest (100%) similarity suggesting that they might originate from the same clonal lineage.

## 4. Discussion

Microsatellite markers distinguish themselves as codominant, multiallelic, highly polymorphic genetic markers, requiring small amounts of DNA for straightforward PCR and gel electrophoresis analysis [32] .

In the current research, di, tri, tetra nucleotide repeat primers were used to obtain multilocus profiles using a MP-PCR assay for the evaluation of relative abundance, nature and polymorphism among 16 different common phytopathogenic fungal species. Also, multilocus profiles obtained in *F. graminearum*, *F. culmorum*, and *Mycosphaerella graminicola* as models for plant pathogens were evaluated. (ATG)5 amplification provided better discrimination of intraspecific diversity with *M. graminicola* strains, but we assumed that these may arise through primer competition, differential efficacy of primer extension at the chosen annealing temperature, in contrast to what could happen with the MP-PCR technique. Our results revealed a high degree of genetic diversity in the *M. graminicola* population in Germany.

The polymorphism found using primer (ATG)5 distinguished different patterns among the analyzed *M. graminicola* isolates, even though in some cases based in some faint bands. Thus, their application would be more inclined to diagnosis than to population genetics studies. The usual method to develop microsatellites involved hybridization-based identification of genomic clones containing possible nucleotide repeats, sequencing of the clones and designing primers that flank the repeat region. This strategy can be effective, but is costly in resources and time. Its use in the genus *Mycosphaerella* resulted in the identification of only nine microsatellites in *M. graminicola* [33]. Highly polymorphic microsatellite markers that can be multiplexed for high-throughput genetic analyses of *M. graminicola* and related species [34].

The highest number of polymorphic DNA fragments were produced using ISSR primers (ATC)_7_ and (GTG)_5_, which detected bands in 38 *M. graminicola* isolates [35]. Nine pairs of single-locus microsatellite primers were used to analyze the genomic DNA of 90 isolates of *M. graminicola* that were collected using a hierarchical sampling procedure from different locations, leaves, and lesions within a wheat field near Saskatoon. Allelic series at eight different loci were detected [36]. Eight single-locus microsatellite markers were used to study genetic variability in a German population of *M. graminicola*. The results showed that there was a high level of genetic variability within the population. The genetic diversity detected within the *M. graminicola* population and the pattern of the distribution of genetic variability suggests that sexual reproduction occurs in the population. Chen and McDonald [37] reported that sexual reproduction played a major role in the genetic structure of populations of *M. graminicola* in the United States. This hypothesis is supported by the recent discovery of the sexual stage of this pathogen in Manitoba [38]. Of the 20 primers tested, only (TGG)5, (GTA)5 and (GGTT)4 were found to produce non polymorphic bands from either *F. graminearum* or *F. culmorum* while (AG)7C, (TTTC)4 and (TGT)5 amplified 100% of polymorphic bands. Microsatellite primer PCR (MP-PCR) yielded highly reproducible and complex genomic fingerprints, with several bands ranging in size from 200 to 3000 bp. Of the 20 primers tested, only (TAGG)4, (TCC)5 and (CA)7T produced a high number of polymorphic bands from either *F. graminearum* or *F. culmorum*. Based on the specific PCR fingerprints and the high interspecies variation of these banding patterns, a clear distinction between all species was possible. Among trinucleotide repeats, 53 different types of repeat motifs were identified and the CTT repeat motif was predominant in the *F. graminearum* genome [39]. Microsatellite-primed polymerase chain reaction using the dinucleotide and tetranucleotide primers showed clear polymorphisms among the different *Fusarium* spp. isolates. Microsatellite-primed PCR fingerprinting with (GACA)_4_ primer discriminated between *C. naterciae*, *C. radicalis*, and *C. parasitica* [40].

Both primers gave similar results in phenetic analysis of genetic similarity between populations [24]. Of all the primers tested, only the microsatellite repeats (CA)8 and (GACA)4 were unable to generate visible DNA fingerprints. It is possible that these repeats are not present in the genomes of these fungi. Another possibility is that the primer annealing sites are at such a distance that amplification by Taq polymerase is impossible. These microsatellites will be useful in population genetic studies of these fungi. The results presented herein indicated that a microsatellite technique provides an efficient tool for the identification of polymorphic loci that can be used to monitor the genetic differences between phytopathogenic fungi. Upcoming research is warranted to develop more microsatellite primers with a wider array of *Fusarium* spp.

## 5. Conclusions

Twenty two microsatellite primers presented here will be valuable for future investigations of the population structure of plant pathogenic fungi. Considering a considerable diagnostic value of markers developed in the current study, they might be a useful tool for screening large fungal collections and generating fingerprints patterns convenient to cluster isolates into specific groups. It could be applied for differentiating individual isolates from a clonal lineage. It will also be useful to characterize undefined collections in order to group isolates into potential species groups.

## Figures and Tables

**Table 1:** Fungal species and isolates used in this study.

Fungal species	Isolates	Abbrevation	Host	Origin
1. *Mycosphaella graminicola*	K-Or-1	Mg	Wheat	Germany
2.	K-Or-30		Wheat	Kiel, Germany
3.	K-Or-38		Wheat	Kiel, Germany
4.	K-Or-44		Wheat	Kiel, Germany
5.	OK-102		Wheat	Kiel, Germany
6.	OK-108		Wheat	Kiel, Germany
7.	OK-109		Wheat	Kiel, Germany
8.	OK-112		Wheat	Kiel, Germany
9.	OK-120		Wheat	Kiel, Germany
10.	K-Ba-10		Wheat	Kiel, Germany
11.	K-Ba-20		Wheat	Kiel, Germany
12.	K-Ba-30		Wheat	Kiel, Germany
13.	K-Ba-40		Wheat	Kiel, Germany
14.	K-Ba-60		Wheat	Kiel, Germany
15.	G-Or-1		Wheat	Kiel, Germany
16.	G-Or-6		Wheat	Göttingen, Germany
17.	G-Or-8		Wheat	Göttingen, Germany
18.	G-Or-88		Wheat	Göttingen, Germany
19.	G-Or-102		Wheat	Göttingen;Germany
*Fusarium oxysporum f. sp. vasinfectum*		Fov	Cotton	Egypt
*F. solani*		Fs	Cotton	Egypt
*F. germanium*	Isolate 37	Fg	Wheat	Germany
*F. culmorum*		Fcu	Wheat	Germany
*F. poae*		Fp	Wheat	Germany
*Macrophomina phaseolina*		Mp	Cotton	Egypt
*Trichoderma harizinum*		Th	Cotton	Egypt
*Septoria tritici*		St	Wheat	Germany
*Stagonospora nodorum*	GB1	Sn	Wheat	Germany
*Pyrenophora teres*		Pte	Barley	Germany
*Pyrenophora tritici-*repentis.	Greifenhagen I	Ptr	Wheat	Germany
*Pseudocercosporella herpotrichoides*		Ph	Wheat	Germany
*Penicillium* sp.		Pen	Wheat	Germany
*Alternaria* sp.		Alt	Wheat	Germany
*Cercospora beticola*		Cb	Sugerbeet	Germany
*Chaetomium* sp.		Ch	Cotton	Egypt

**Table 2:** Microsatellite primers developed in this study and their length, GC content, molecular weight, motifs, optimum annealing temperature, size range of fragments and mean number of alleles amplified from the 16 fungal species amplified.

Nucleotides repeats	Primers	Motif	Length	GC Content (%)	MW	Optimum AT (°C)	Range of fragment size (bp)
Di	(CT)7G	CT	15	53.3	4420.9	40	400–2000
	(CA)7T	CA	15	46.7	4458.9	52	300–1700
	(AG)7C	AG	15	53.3	4724.1	48	200–2500

Tri	(CTG)5	CTG	15	66.7	4550.9	52	450–2000
	(AGG)5	AGG	15	66.7	4796.1	52	350–2000
	(TCC)5	TCC	15	66.7	4350.8	50	400–2000
	(ACG)5	ACG	15	66.7	4596.0	50	400–2000
	(TGG)5	TGG	15	66.7	4751.1	50	350–1500
	(GTA)5	GTA	15	33.3	4671.1	40	600–2000
	(ATG)5	ATG	15	33.3	4671.1	50	200–3000
	(TAC)5	TAC	15	33.3	4470.9	40	700–1500
	(TGC)5	TGC	15	66.7	4550.9	40	1000–1800
	(GCT)5	GCT	15	66.7	4550.9	52	600–2000
	(TGT)5	TGT	15	33.3	4626.0	40	400–1800

Tetra	(TGAC)4	TGAC	16	50.0	4881.2	55	400–2500
	(GTCA)4	GTCA	16	50.0	4881.2	50	400–1800
	(TAGG)4	TAGG	16	50.0	5041.3	50	200–2000
	(TTTC)4	TTTC	16	25.0	4745.1	40	300–2000
	(TACC)4	TACC	16	50.0	4721.1	45	550–2000
	(GGTT)4	GGTT	16	50.0	5005.3	40	550–2500

**Table 3:** Total number of bands amplified from sixteen fungal species with 20 newly developed microsatellite primers (MP) and five known primers.

MP Primers	Number of alleles amplified from different fungal species																Total No. of Band	Mean alleles amplified per species

	Fov	Fs	Fg	Fcu	Fp	Mp	Th	St	Sn	Pte	Ptri	Ph	Pen	Alt	Cb	Ch		
(CT)7G	2	4	4	3	5	0	1	3	5	3	3	2	4	2	0	3	43	2.7
(CA)7T	4	3	7	4	23	5	3	3	4	3	2	3	4	4	5	4	61	3.8
(AG)7C	1	2	2	2	3	4	3	6	0	4	6	2	4	8	3	5	52	3.3
(CTG)5	4	4	4	4	5	4	4	5	4	6	4	4	5	4	7	3	71	4.4
(AGG)5	10	8	8	7	8	5	6	4	8	6	4	6	5	5	6	7	103	6.4
(TCC)5	8	8	9	9	4	5	4	6	5	4	6	5	6	5	3	3	90	5.6
(ACG)5	6	6	4	6	7	6	5	7	10	7	8	7	7	7	7	7	107	6.7
(TGG)5	3	5	5	5	7	6	4	6	7	5	7	5	6	6	6	8	91	5.7
(GTA)5	0	0	0	0	2	0	0	3	0	3	3	0	3	0	3	0	17	1.1
(ATG)5	4	5	6	2	7	3	4	5	1	3	4	4	5	2	4	4	63	3.9
(TAC)5	0	0	0	0	0	0	0	3	0	0	0	0	0	0	0	0	3	0.2
(TGC)5	3	3	1	2	3	0	0	3	2	5	1	3	3	0	3	0	32	2.0
(GCT)5	3	2	4	3	3	0	1	2	1	2	3	3	2	2	4	3	38	2.4
(TGT)5	0	1	1	0	0	0	2	0	0	0	0	1	1	2	1	0	9	0.6
(TGAC)4	6	3	6	4	6	4	1	1	6	5	4	4	5	8	2	2	67	4.2
(GTCA)4	1	2	4	5	2	4	2	4	3	2	2	5	3	2	2	1	44	2.8
(TAGG)4	4	4	8	3	4	3	2	3	4	3	5	3	5	3	5	3	62	3.8
(TTTC)4	3	1	3	1	3	2	1	2	4	3	2	2	4	2	2	3	38	2.4
(TACC)4	5	4	3	2	4	0	0	4	4	1	2	3	4	7	2	3	48	3.0
(GGTT)4	1	3	1	1	4	0	0	4	5	2	2	2	2	5	1	3	36	2.3
T3B	5	5	7	7	10	6	7	8	9	7	6	5	4	5	4	6	105	6.6
M13	4	4	6	5	4	4	2	2	6	4	4	3	5	7	5	4	69	4.3
(GTG)5	5	6	6	5	8	4	6	4	4	4	4	6	8	8	8	4	90	5.7
(GTGC)4	6	5	3	6	2	4	4	2	1	3	4	5	6	4	4	3	62	3.9
(CAG)3	4	5	5	4	5	5	5	6	4	4	5	5	4	5	4	5	75	4.7

(TB) total number of bands, * T3B. (5′-AggTCgCgggTTCgAATCC-3′)M13. (5′-gAgggTggCggTTCT-3′).

**Table 4:** Comparison of total number of bands (TB), number of polymorphic bands (PB), percentage of polymorphic bands (PB %) amplified from *Fusarium graminearum* (Fg) and *F. culmorum* (Fcu).

Primers	Total bands			Polymorphic bands			PB%

	Fg	Fcu	Total	Fg	Fcu	Total	
(CT)7G	4	3	7	2	1	3	42.9
(CA)7 T	7	4	11	4	1	5	45.5
(AG)7C	2	2	4	2	2	4	100.0
(CTG)5	4	4	8	2	2	4	50.0
(AGG)5	8	7	15	2	2	4	26.7
(TCC)5	9	9	18	4	3	7	38.9
(ACG)5	4	6	10	0	2	2	33.3
(TGG)5	5	5	10	0	0	0	0
(GTA)5	0	0	0	0	0	0	0
(ATG)5	6	2	8	3	0	3	37.5
(TAC)5	0	0	0	0	0	0	0
(TGC)5	1	2	3	1	1	2	67.7
(GCT)5	4	3	7	2	0	2	28.6
(TGT)5	1	0	1	1	0	1	100.0
(TGAC)4	6	4	10	3	1	4	40.0
(GTCA)4	4	5	9	2	2	4	44.4
(TAGG)4	8	3	11	6	2	8	72.7
(TTTC)4	3	1	4	3	1	4	100
(TACC)4	3	2	5	2	1	1	40.0
(GGTT)4	1	1	2	0	0	0	0
*T3B	7	7	14	2	2	4	28.6
*M13	6	5	11	1	1	2	18.2
(GTG)5	6	5	11	1	0	1	9.09
(GTGC)4	3	6	9	1	4	5	55.6
(CAG)3	5	4	9	2	0	2	22.2

* T3B. (5′-AggTCgCgggTTCgAATCC-3′)M13. (5′-gAgggTggCggTTCT-3′).
